# Socioeconomic inequalities in children’s diet: the role of the home food environment

**DOI:** 10.1186/1479-5868-12-S1-S4

**Published:** 2015-07-27

**Authors:** Nalini Ranjit, Anna V Wilkinson, Leslie M Lytle, Alexandra E Evans, Debra Saxton, Deanna M Hoelscher

**Affiliations:** 1 Michael & Susan Dell Center for Healthy Living, The University of Texas School of Public Health Austin Regional Campus, 1616 Guadalupe St., Austin, TX 78701, USA; 2 Gillings School of Global Public Health, 135 Dauer Drive, Chapel Hill, NC 27599-7400, USA; 3 Texas Department of State Health Services, Division for Family and Community Health Services, PO Box 149347, Austin, TX 78714-9347, USA

**Keywords:** socioeconomic disparities, healthy eating, home food environment, neighborhood

## Abstract

**Background:**

It is well documented in the literature that low socioeconomic status (SES) is associated with lower consumption of healthy foods and that these differences in consumption patterns are influenced by neighborhood food environments. Less understood is the role that SES differences in physical and social aspects of the home food environment play in consumption patterns.

**Methods:**

Using data on 4^th^ grade children from the 2009–2011 Texas School Physical Activity and Nutrition (SPAN) study, we used mixed-effects regression models to test the magnitude of differences in the SPAN Health Eating Index (SHEI) by parental education as an indicator of SES, and the extent to which adjusting for measures of the home food environment, and measures of the neighborhood environment accounted for these SES differences.

**Results:**

Small but significant differences in children’s SHEI by SES strata exist (-1.33 between highest and lowest SES categories, p<0.01). However, incorporating home food environment and neighborhood environment measures in this model eliminates these differences (-0.7, p=0.145). Home food environment explains a greater portion of the difference. Both social (mealtime structure) and physical aspects (food availability) of the home food environment are strongly associated with consumption of healthy and unhealthy foods.

**Conclusions:**

Our findings suggest that modifiable parent behaviors at home can improve children’s eating habits and that the neighborhood may impact diet in ways other than through access to healthy food.

## Background

Socioeconomic inequalities in diet, nutrition and dietary patterns across the life span have been demonstrated in a number of developed countries [1-8]. Several recent reviews confirm a consistent positive association between multiple indicators of socioeconomic status (SES) and micronutrient intake as well as multiple dietary constituents [9-11]. While most of these studies were focused on adults, low SES was also found to be associated with poor dietary practices in an extensive review focused on health behaviors among adolescents [12]. This association appears to hold across multiple indicators of socioeconomic status, including education, income and occupational class [13]. In the United States, literature focused on traditional SES indicators is relatively limited, but a number of studies have documented racial and ethnic differentials in dietary practices and adherence to dietary guidelines of both children and adults [14-17]. Again, it is likely that socioeconomic factors explain at least some part of the observed racial and ethnic differences in diet in the U.S. [18]. Across these studies, socioeconomic disadvantage is consistently associated with lower consumption of fruits and vegetables and higher consumption of energy-dense foods [9-11]. Inequalities in diet are important to understand because they are a key contributor to inequalities in obesity as well as in overall health [19-21].

A recurring theme in studies of both racial and socioeconomic disparities in diet is the role of physical attributes of the built environment, particularly neighborhood influences with regard to grocery options and advertising [22]. Neighborhoods in the U.S. and in other developed countries serve as a stratifying mechanism, sorting racial and ethnic minorities as well as economically disadvantaged groups into distinct and segregated social spaces. The food environments of these disadvantaged neighborhoods are typically characterized by limited access to retail outlets selling affordable healthy foods such as fresh fruits and vegetables, as well as by a high density of fast food outlets and convenience stores where processed and energy-dense foods are readily available [23-28]. There is also evidence that individuals living in low-income neighborhoods are subject to a greater degree of outdoor food advertising, which further exacerbates SES differences in food preferences and diet [29]. A parallel, but smaller, literature explores the role of schools. Schools, like neighborhoods, are often subject to differentiated environmental characteristics by socioeconomic status [30,31]; for instance, the percentage of low-income students in schools is a reliable predictor of the number of fast-food outlets and convenience stores located close to the schools [32].

In contrast to the extensive literature on the neighborhood environment and its role in engendering socioeconomic differences in diet, few studies have examined if socioeconomic differences in the home food environment play a role in SES disparities in healthy versus unhealthy diets. This gap in the literature is particularly notable considering the large body of work that has identified different aspects of the home food environment and how these factors are related to the diets of children. Indeed, some research suggests that the home food environment may play a role independent of the neighborhood food environment [33]. Both physical and social aspects of the home food environment appear to be related to what children eat [34]. Relevant physical aspects of the home food environment include the availability, accessibility and meal portion sizes. Beyond these physical aspects of food at home, a variety of aspects of mealtime structure, including whether families eat together (family meals), whether they watch television during meals, where the meal is prepared (restaurant or home), and parental modeling appear to influence children’s dietary behavior [35-43]. A number of studies have demonstrated that several of these aspects of the home food environment appear to show distinct socioeconomic patterning, with lower SES homes characterized by generally more obesogenic home food environments as well as unhealthy eating [44-47]. Yet there is little published work evaluating the extent to which socioeconomic differences in the home food environment explain overall socioeconomic differences in the diet of children. In particular, it is important to determine if the social aspects (rather than the physical resource availability) of the home food environment play any role in disparities in children’s diets after accounting for neighborhood. Such a determination would uncover potentially modifiable behaviors in the home environment that are associated with children’s diets and diet disparities.

In this paper, we attempt to address this gap in the literature by examining these associations in elementary school children. Our hypotheses are as follows: (1) a socioeconomic gradient exists in the consumption of healthy and unhealthy foods by elementary school children; (2) adjusting for physical and social aspects of the home food environment reduces the magnitude of socioeconomic differences in such consumption; and (3) adjusting for measures of the neighborhood food environment should also attenuate socioeconomic differences in consumption. Data for our study are drawn from a statewide survey of 4^th^-grade students in Texas, USA. Texas, the second most populous state in the U.S., is among the most diverse. Demographic trends in Texas are considered to presage demographic trends of the nation; hence, lessons learned in this context are likely to have national relevance [48].

## Methods

### Population and survey instrument

To address the above hypotheses, we used data from the 2009–2011 Texas School Physical Activity and Nutrition (SPAN) surveillance study. SPAN is a periodic, cross-sectional survey of obesity, diet and physical activity behaviors among Texas public school students in the 4th, 8th and 11th grades. In 2009–2011, for the first time, surveys of 4^th^-grade children were supplemented with surveys for their parents. The SPAN parent survey is complementary to the 4^th^-grade student survey and assesses detailed household demographics, parent influence on family nutrition and physical activity, and attributes of the home, family and child that cannot be readily obtained from 4^th^ graders. Analyses for this report are restricted to 3,131 4^th^ graders (62% of the 5,035 4^th^-grade children surveyed) for whom matched parent surveys were available.

SPAN student surveys were administered in school classrooms following a standard protocol, and both parental consent and child assent were obtained. Parent surveys were administered as take-home surveys, and were available in both Spanish and English. Full details on the SPAN study have been published previously [49]. The Institutional Review Board (IRB) of The University of Texas Health Science Center at Houston, the Texas Department of State Health Services IRB, and participating school districts each provided approval for the SPAN study.

### Measures

Measures utilized to examine these associations are summarized in Table 1 and further described below.

**Table 1:** Summary of measures used

Name of measure	Description	Details	Range
SPAN Healthy Eating Index (SHEI)	Combines information on previous day consumption of both healthy marker foods, and unhealthy marker foods, as reported by child	Eight healthy foods included in the index include baked or grilled (not fried) meats, milk, yogurt, brown rice, brown pasta, a variety of vegetable types, fruits (not fruit juice), and beans. The five unhealthy foods include fried meat, red meat, sugar-sweetened beverages, salty fried snacks, and a variety of dessert items. Items are summed, and scaled to a range of 0-100, with higher values representing healthier diets.	0-100
Socioeconomic Status	Categorical measure of parental education	Single measure classified into 4 categories	0-3
Neighborhood Environment (specific)	Perceived Neighborhood Food Access (reported by parents)	Single binary measure	0-1
Neighborhood Environment (global)	Neighborhood/School SES measure: Percent of children not eligible for free / subsidized school lunches	Single continuous measure, obtained from publicly available administrative data	0-100
Physical Home food environment index	Consists of 2 sub-indices – availability of healthy foods and absence of unhealthy foods	Availability of healthy food derived as sum of 4 binary indicators describing availability of each of 4 healthy marker foods) Absence of unhealthy food derived as sum of 2 binary indicators describing frequency of serving each of 2 unhealthy marker foods)	0-4 0-2
Social Home food environment index	Includes measures of frequency of family meals, watching TV during meals, and eating at a restaurant in the past week as reported by the child	Three binary measures are summed into a single index describing the social home food environment	0-3
Socio-demographic measures	Age of child Number of children in household Ethnicity Location: Urban, rural or suburban	Four different measures used and entered into models as distinct measures.	

#### Outcome measures

The primary outcome measure was the SPAN Healthy Eating Index (SHEI), constructed from diet measures available on the survey as 22 detailed questions included on the child survey, which referenced specific marker foods or food groups. The questions pertained to number of times each food group was consumed on the previous day and responses ranged from none (0) to three or more. These items have earlier been shown to have good to excellent reproducibility among 4^th^-grade children, as assessed by Spearman rank order correlations and κ statistics [50]. The SHEI is a *composite* measure comprising *both healthy and unhealthy items*. Responses to items asking about previous day consumption of baked or grilled (not fried) meats, milk, yogurt, brown rice, brown pasta, a variety of vegetable types, fruits (not fruit juice), and beans were summed. Responses to items querying frequency of consumption of fried meat, red meat, sugar-sweetened beverages, salty fried snacks, and a variety of dessert items were reverse coded, so that the lowest frequency represented the healthiest eating practice. These two sums were combined into the single composite SHEI and rescaled from 0–100. In addition, sub-analyses examined individual healthy and unhealthy food groups of interest separately. Items included in these two broad classes were: (1) fruits and vegetables, (2) sugar-sweetened beverage consumption, (3) consumption of salty snacks, (4) milk, and (5) desserts.

#### Exposures, pathway variables and potential confounders

##### Socioeconomic status

Education of the responding parent, obtained in 5 ordinal categories, was used as the sole measure of individual socioeconomic status. Education is the most commonly used measure of socioeconomic status in studies of health outcomes, and besides being less subject to misreporting, may also be a more consistent measure of long-term SES than either occupation or wealth [51]. *parental education* was categorized into 4 levels as follows: (1) less than high school (2) high school or equivalent completed (3) some college education but no degree obtained, and (4) one or more college degrees obtained.

##### Neighborhood environment

Two measures, one specific and one global, were used to construct measures of the neighborhood food environment. For the specific *Neighborhood Food Access* measure, parents were asked to indicate on a 5-point scale the degree to which they perceived access to healthy food to be a problem in their neighborhood, with options ranging from “not a problem” to “a severe problem.” This was dichotomized so that a score of 1 represented not having a problem with access. In addition to this measure of perceived access to healthy food, a more global measure of the neighborhood environment, the *Neighborhood / School SES* measure was constructed using objective, publicly available information (published by the Texas Education Agency) on the percent of children eligible for free or subsidized lunch in the school attended by their child. Because of zoning laws in the United States, children in public schools usually attend schools within a restricted area around their homes; thus, the smaller catchment area of the school district serves as a reasonable proxy for a neighborhood of residence, and is frequently used as a proxy measure for individual SES [52-54]. This measure was reverse coded so that the highest score of 100 represented the highest school SES. For analyses involving use of the neighborhood environment, both of these measures were included in models as separate variables.

##### Home food environment

Data from both the parent and child questionnaires were used to construct social and physical indices of the home food environment. Physical measures available on the survey include the following: (a) *Availability of healthy food*: Parents were asked how many times in the past week the following foods had been served during meals: fruits, vegetables, milk, and whole grain products. Each of these items had seven response categories, ranging from “did not serve” to “served 7 or more times.” Responses to these questions were collapsed into binary measures (3 or more vs. fewer than 3 times) and summed into a 5-point scale (range: 0–4). (b) *Absence of unhealthy food*: Similar questions ascertained whether or not sugar-sweetened cereals were served at breakfast in the past week, and whether or not sugar-sweetened beverages were served during meals. Responses were reversed and summed into a 3-point scale (0–2), where higher scores imply lower availability of these foods. While this is not a comprehensive measure of unhealthy food availability, sugar-sweetened beverages serve as a good proxy measure of consumption of other unhealthy foods [52]. The *Physical home food environment* index was derived as the sum of these two availability measures. Availability measures used in the literature usually refer to availability in the food pantry; by extending the questions to availability at meals, we are able to incorporate elements of parental practices around eating. Social measures included: (a) *Family meals*: this was a binary measure, given a value of 1 if parents reported that they ate a sit-down meal with their child 3 or more times in the past week. (b) *Television watching during meals*: a parent report of watching TV during dinner 3 or more times in the past week was taken as a proxy of TV during dinner for children as well, and coded as 1 on a binary measure. (c) *Eating at a restaurant*: this binary variable was based on the child’s response to the question “Yesterday, how many times did you eat at a sit-down restaurant?” A similar parent measure could not be utilized as there were too many missing values, and the wording made it unclear if it was the parent eating at restaurants, or if it was the child. These three measures were combined into a single *Social Home Food Environment* index measure. Analyses involving the home food environment include both the physical and social home food environment measures, and in some cases, their component measures.

#### Potential confounders

Demographic measures that were deemed potential confounders were examined for their association with education, and retained if they showed significant associations (i.e., p<0.05). These included measures of the age of the child, number of children at home (coded as <=2, 3-4, and >=5), ethnicity (coded as Hispanic, non-Hispanic Black, and all other ethnic / racial groups including non-Hispanic White), and whether the child resides in a rural, urban, or other urban/suburban location. Information on all these measures except location was obtained from parent surveys; location was derived from the location of the school district as used in the sampling plan for SPAN. Number of kids and age were not included in the final models, as they were not associated with any of the outcomes.

### Statistical methods

After conducting a descriptive analysis of important demographic and socioeconomic characteristics of the subjects, mixed-effects regression models were tested to examine associations of interest. The SPAN Healthy Eating Index (SHEI) was the primary outcome of interest. Regressions were used to examine, first, the magnitude of differences in the SHEI by parental education, and second, the extent to which adjustment for the home food environment and the neighborhood environment explained these differences. Models were adjusted for race/ethnicity and rural / urban location. To account for possible school-level clustering of outcomes, models included a random intercept at the school level. An identity covariance structure was assumed, and estimation was by maximum-likelihood. Additional analyses were done to allow detailed examination of the role of the home food environment. Associations of individual components of the physical and social home food environment measures with the SHEI, as well separately with important healthy and unhealthy food groups, were examined with regression analyses adjusted for race/ethnicity and a school level random effect. All analyses were carried out using SAS 9.2 (SAS Institute, Research Triangle, NC).

## Results

Analyses were restricted to 3,001 parent-child dyads that had information available on parental education. Table 2 shows the socio-demographic composition of the children in the sample, by level of parental education. The mean age of the children was 9.6; although there was a significant declining trend in child age across parental education levels (p<0.001), the differences across categories were small. No gender differences were evident across education levels. About half the sample was Hispanic; Blacks comprised only 12% of this population, which is comparable to the percentage of Blacks in Texas. Over a quarter of Hispanic parents represented here did not complete high school, and fewer than 1 in 5 had a college degree, compared to Blacks, with 37% college educated, and White/others, with over 50% with a college degree. A plurality of the sample (48%) reported 1–2 children at home, closely followed by 43% with 3–4 children at home. The number of children at home is inversely related to the level of parental education. About half of the population represented in the sample resided in rural areas, while 31% resided in suburban areas. In all, about two-thirds of the children were economically disadvantaged, on average, in each school. The school socioeconomic measure clearly is associated with the educational level of the parent.

**Table 2:** Socioeconomic characteristics of children, by level of parental education

		*Parental education*				
	Full Sample	Less than high school	High school/GED	Some college	College degree	*p for difference*
						
Number of children	3001	450	733	815	1003	
Mean (SD) age	9.57	9.62 (0.65)	9.62 (0.64)	9.59 (0.6)	9.5 (0.55)	*<.0001*
						
Gender						
Boy	1403 (0.47)	14.04	23.73	26.87	35.35	
Girl	1598 (0.53)	15.83	25.03	27.41	31.73	*0.1625*
						
Race/Ethnicity						
Black	357 (0.12)	5.32	26.33	31.65	36.69	
Hispanic	1490 (0.5)	25.84	31.34	23.76	19.06	
White / other	1154 (0.38)	3.99	14.9	30.16	50.95	*<.0001*
						
Number of kids at home						
1-2 kids	1417 (0.48)	9.1	21.38	29.78	39.73	
3-4 kids	1280 (0.43)	18.13	27.34	24.77	29.77	
5 or more kids	250 (0.08)	25.6	27.6	26.8	20	*<.0001*
						
Rural/ Urban classification						
Rural	1524 (0.51)	14.44	24.8	27.76	33.01	
Suburban	924 (0.31)	17.32	21.21	24.68	36.8	
Urban	553 (0.18)	12.66	28.75	29.66	28.93	*0.0005*
						
% socioeconomically disadvantaged	66.6	84.9	76.8	64.6	51.5	

In Table 3, we examine the magnitude of differences in the SHEI across levels of parental education, and the extent to which such differences are explained by aspects of the neighborhood and home food environment. These differences are explored by way of four models. Model 1 estimates SHEI scores across levels of parental education after adjustment for race/ethnicity and rural/urban/suburban status. Model 2 examines these scores after further adjustment of Model 1 for neighborhood environment (perceived food access, and neighborhood/school SES); Model 3 adds measures of the physical and social aspects of the home food environment. Model 4 is a composite model, adjusting for both neighborhood and home food environment.

**Table 3:** Estimated Healthy Eating Index value by level of parent education, before and after adjusting for neighborhood and home food environment

	**Model 1 (estimated mean HEI, 95% CI)**	**Model 2 (estimated mean HEI, 95% CI)**	**Model 3 (estimated mean HEI, 95% CI)**	**Model 4 (estimated mean HEI, 95% CI)**
Education				
< high school	38.08 (37.3, 38.9)	38.23 (37.4, 39.1)	38.39 (37.6, 39.2)	38.52 (37.7, 39.4)
High school/GED	37.96 (37.3, 38.6)	38 (37.3, 38.7)	38.2 (37.6, 38.9)	38.23 (37.6, 38.9)
Some college	38.08 (37.5, 38.7)	38.07 (37.5, 38.7)	38.09 (37.5, 38.7)	38.09 (37.5, 38.7)
College degree	39.41 (38.8, 40)	39.33 (38.7, 39.9)	39.27 (38.7, 39.9)	39.22 (38.6, 39.8)
				
Difference in SHEI between lowest and highest educational category	-1.33 (-2.24, -0.42)	-1.1 (-2.04, -0.15)	-0.87 (-1.78, 0.04)	-0.7 (-1.65, 0.24)
*p for difference*	*0.0041*	*0.0227*	*0.0606*	*0.145*

NOTES: SPAN Healthy Eating Index (SHEI) is a composite measure combining responses to consumption of healthy foods and unhealthy foods, scaled to 100, and coded so that higher scores represent healthier diets. See Table 1 for a listing of the healthy and unhealthy foods comprising the SHEI. Table 3 presents regression-derived mean values of the SHEI for each of the educational categories, as well as the contrast between the highest and lowest categories of education. Model 1 examines differences in children’s SHEI scores across levels of parental education after adjusting for race/ethnicity and rural/urban location. Model 2 adds neighborhood environment measures to Model 1; Model 3 adds home food environment measures to Model 1. Model 4 is the full model, and adds both neighborhood and home food environment measures to Model 1.

The estimates suggest that adjusting for either neighborhood or home food environment reduces the magnitude of the educational gap, from -1.33 (p=0.004) to -1.1 (p=0.022) and to -0.87 (p=0.0606) respectively. In the final model, with both neighborhood and home food environment measures included, the SHEI gap across the index educational categories falls to -0.7, and is no longer significant. Although the measures for neighborhood food environment and home food environment are not of comparable magnitude, the small change in the educational gap from Models 1 to 2 suggests neighborhood location explains very little of the variation in healthy eating by parental education. Comparison of Model 1 and Model 3 estimates, on the other hand, suggests that physical and social aspects of the home food environment explain a considerable portion of the educational gap in healthy eating. In terms of the association of individual variables (parameter estimates not shown), the school SES measure was significantly and positively associated with the SHEI, but perceived access to healthy food in the neighborhood was not associated. This suggests that the effect of neighborhood SES is independent of parental education. Both the physical and social aspects of home food environment were significantly associated with SHEI in the expected direction. Regression parameters also indicated significantly lower SHEI scores for Black children, and children from urban areas. In all 4 models examined, the magnitude of these gaps exceeded the gaps across the highest and lowest categories of parental education.

The impact of individual components of the home food environment on SES associations with children’s diets is detailed in Table 4. All models are adjusted for education, race/ethnicity, rural/urban location, and neighborhood environment. Separate models are estimated for the composite SHEI, for healthy foods alone, and for unhealthy foods alone to determine if separate aspects of the home environment are associated differently with these food types. Each of the home food environment measures examined is significantly associated with the SHEI, consumption of healthy foods, and consumption of unhealthy foods, except for the variable measuring family meals, which is not associated with any of these outcomes. Not watching TV during dinner is associated with significantly greater consumption of healthy foods (0.26), and significantly lower consumption of unhealthy foods (-0.27). Estimates for the restaurant measure are surprising. Not eating at a restaurant is associated with significantly lower consumption of unhealthy foods (-2.11) as well as healthy foods (-1.18). Eating at a restaurant appears to be associated with greater intake of food in general, both healthy and unhealthy. Availability of healthy foods at the dinner table is associated with greater intake of healthy foods, while restriction of unhealthy foods reduces consumption of unhealthy foods. The inclusion of home food environment measures in models examining SES associations with food appears to primarily explain consumption of unhealthy foods, with the contrast between highest and lowest parental education categories decreasing from 0.88 to 0.44. No such change in the contrast is evident in the model for healthy foods alone.

**Table 4:** Associations of individual components of a healthy food environment with healthy and unhealthy eating

	**Estimated association with composite Healthy Eating Index**		**Estimated association with healthy foods**		**Estimated association with unhealthy foods**	
	Beta (95% CI)	*p-value*	Beta (95% CI)	*p-value*	Beta (95% CI)	*p-value*
Regular family meals (REF: No)	0.13 (-0. 82, 1.07)	*0.7944*	-0.08 (-0.66, 0.51)	*0.8008*	0.05 (-0.37, 0.46)	*0.8299*
						
TV is not on during dinner (REF: TV is on)	0.8 (0.24, 1.36)	*0.0052*	0.26 (-0.09, 0.61)	*0.1429*	-0.27 (-0.51, -.02)	*0.0349*
						
Child did not eat at a restaurant on the previous day (REF: Had at least one restaurant meal)	1.46 (0.73, 2.2)	*<.0001*	-1.18 (-1.64, -0.73)	*<.0001*	-2.11 (-2.44, -1.79)	*<.0001*
						
Availability of healthy foods at the family dinner table (range: 0-4)	0.76 (0.54, 0.98)	*<.0001*	0.38 (0.24, 0.51)	*<.0001*	-0.15 (-0.25, -0.06)	*0.0019*
						
Restriction of unhealthy foods at the family dinner table (range: 0-2)	0.35 (-0.01, 0.71)	*0.0590*	-0.06 (-0. 29, 0.16)	*0.5786*	-0.25 (-0.41, -0.09)	*0.0021*
						
Difference between lowest and highest educational category, *before* adjusting for home food environment	-1.1 (-2.04, -0.15)	*0.0227*	0.17 (-0.42, 0.76)	*0.5693*	0.88 (0.46, 1.31)	*<.0001*
Difference between lowest and highest educational category, *after* adjusting for home food environment	-1.04 (-2.01, -0.07)	*0.0356*	0.17 (-0.44, 0.77)	*0.5895*	0.84 (0.41, 1.27)	*0.0001*

NOTES: SPAN Healthy Eating Index (SHEI) is a composite measure combining responses to consumption of healthy foods and unhealthy foods, scaled to 100, and coded so that higher scores represent healthier diets. See Table 1 for a listing of the healthy and unhealthy foods comprising the SHEI. This table examines the composite SHEI, as well as the healthy foods and unhealthy foods components separately.

## Conclusions

In these cross-sectional analyses of a large sample of elementary school-age children in Texas, we examined both socioeconomic differences (as indexed by parent’s education level) in children’s diet as well as the role of neighborhood and home food environments in explaining those differences. We showed that (a) there exist small but significant differences in children’s diet quality across parents’ education levels, and (b) after accounting for the home food environment, these differences are reduced to insignificance, particularly differences pertaining to consumption of unhealthy foods.

Both social (mealtime structure) and physical aspects (food availability) of the home food environment were strongly associated with consumption of healthy and unhealthy foods, and each of these measures continued to have a strong independent association with diet, even in adjusted models that considered multiple measures of socioeconomic status, neighborhood environment, and other home food environment measures. These findings have significant implications since they suggest that modifiable behaviors at home, such as making available healthy foods, restricting unhealthy foods, turning off the TV, and avoiding eating at restaurants, could all potentially bring about a substantial improvement in children’s eating habits. The findings on availability are especially consistent. Although it has been argued that the cost, and therefore, the availability of healthy foods could explain most socioeconomic differences [11], our results suggest otherwise, for two reasons. First, parent-reported access to healthy food was not a factor in children’s diets; and second, it is apparent that restriction of unhealthy foods also served to increase healthy eating, suggesting that the consumption of healthy foods is motivated by factors other than just their availability.

Although published research examining the role of the home food environment in explaining SES differences in diet is scant, our results regarding the independent association of several home food environment measures with children’s diet are consistent with the literature. The only finding that is different from that reported in the literature was the lack of association of regular family meals with children’s consumption of healthy foods. A number of studies have reported that family meals serve to improve children’s diets [55,56], however, these findings are limited to adolescents and may not carry over to younger children. Additionally, it may be the case that families with younger children are more likely to eat together than families with adolescents, regardless of whether meals are healthy or unhealthy. Moreover, there is some evidence that a high prevalence of family meals coexists with otherwise unhealthy food environments among Hispanics [57].

In contrast, we found that a global measure of neighborhood poverty (school SES) appeared to have a negative influence on children’s diets, independent of the influence of parental education. However, we did not find any association between parent-perceived access to healthy foods and children’s diet in our study. Taken together, these findings suggest that the neighborhood may impact diet in ways other than access to healthy food. Indeed, easy access to unhealthy foods is likely a more important influence on children’s diets; however, we lacked the data to confirm this. Indeed, overall, the influence of neighborhood of residence likely reflects a host of other upstream influences, such as cultural and economic factors, on both diet behaviors and the structuring of the home food environment.

This cross-sectional study has obvious limitations, in addition to potential reverse causation. The SPAN survey was an epidemiologic study intended to obtain limited amounts of information on a wide variety of constructs, and was designed to be comprehensible to a wide swath of a multi-ethnic, socioeconomically diverse population. Hence, the measures utilized offer little variability, rather they are simple and often inherently binary. For instance, socioeconomic status is a complex, multidimensional and nuanced attribute [58], and it is unlikely that the four-level parental education measure we used was able capture the entire range of its complexity. Similarly, measures of the neighborhood and home food environments that we used are likely incomplete proxies of SES. Nevertheless, our use of multiple constructs that each has some bearing on SES was intended to address this shortcoming in our data. A second potential limitation is that the analyses are restricted to a single state in the US. While it is true that Texas, with its high Hispanic population, is not necessarily representative of the U.S. population, it is, however, considered a bellwether state, given current population trends. Despite these limitations, the findings appear credible. Although we found only small differences in diet across parent education levels, it should be noted that these differences were detectable despite our use of a rather imprecise SES measure, and despite the fact that the models were already adjusted for racial and urban / rural differences in diet.

The study has several strengths that lend credibility to its findings. First, the data allowed us to examine several measures of the home food environment in the same study, as well as capture some measure of the neighborhood environment. Second, we separately examined healthy and unhealthy food consumption by children, and were able to identify factors unique to each of these diets. Finally, we relied heavily on parent reports and publicly available measures of aspects of the neighborhood and home food environment, both of which are presumably more reliable than child-reported measures. We believe that this study provides a model for future evaluations of socioeconomic disparities in diet, and highlights several associations that deserve more detailed investigation.

## Competing interests

The authors declare that they do not have competing interests.

## Authors’ contributions

NR conceptualized the manuscript, conducted the analysis, and prepared the first draft of the manuscript. AE and AW participated in conceptualizing the analyses, in examining the results of analysis, and in drafting the paper. DH and DS were instrumental in developing the instruments and obtaining funding for the collection of the SPAN data that were utilized in the analysis. DH, LL, and DS all provided critical review of multiple drafts. Collection of data and the analytical / writing effort on this work were made possible by funding from the Texas Department of State Health Services, as well as a grant from the Michael & Susan Dell Foundation.

## Declarations

This article has been published as part of *International Journal of Behavioral Nutrition and Physical Activity* Volume 12 Supplement 1, 2015: The science of childhood obesity: an individual to societal framework. The full contents of the supplement are available online at http://www.ijbnpa.org/supplements/12/S1. Publication costs for this supplement were funded by the Michael & Susan Dell Foundation.
